# Baseline Ratio of Soluble Fas/FasL Predicts Onset of Pulmonary Hypertension in Elder Patients Undergoing Maintenance Hemodialysis: A Prospective Cohort Study

**DOI:** 10.3389/fphys.2022.847172

**Published:** 2022-03-01

**Authors:** Xiao-Han Ding, Xiaoliang Chai, Jin Zheng, Hong Chang, Wenxue Zheng, Shi-Zhu Bian, Ping Ye

**Affiliations:** ^1^Department of Cardiology, The Second Medical Center and National Clinical Research Center for Geriatric Diseases, Chinese PLA General Hospital, Beijing, China; ^2^Department of Health Care and Geriatrics, The 940^th^ Hospital of Joint Logistics Support of PLA, Lanzhou, China; ^3^Department of Ultrasonography, The 940^th^ Hospital of Joint Logistics Support of PLA, Lanzhou, China; ^4^Department of Cardiology, The 940^th^ Hospital of Joint Logistics Support of PLA, Lanzhou, China; ^5^Institute of Cardiovascular Diseases of Xinqiao Hospital and People’s Liberation Army of China, Chongqing, China; ^6^Department of Cardiology, Xinqiao Hospital, Army Medical University (Third Military Medical University), Chongqing, China

**Keywords:** maintenance hemodialysis, pulmonary hypertension, ESRD, soluble Fas/FasL, risk factors, elder patients

## Abstract

**Background:**

Pulmonary hypertension (PH) is one of the most common complications associated with end-stage renal disease (ESRD). Though numerous risk factors have been founded, other risk factors remain unidentified, particularly in patients undergoing maintenance hemodialysis with elder age. Soluble Fas (sFas) and its ligand FasL (sFasL) have been reported in chronic renal disease patients; however, they have not been identified in the PH patients of elder hemodialysis patients. We aimed to determine the roles of sFas/sFasL in onset of PH in elder patients undergoing maintenance hemodialysis with ESRD.

**Methods:**

Altogether, 163 patients aged 68.00 ± 10.51 years with ESRD who undergoing maintenance hemodialysis in a prospective cohort and were followed-up for a median of 5.5 years. They underwent echocardiography examinations, liver function assessments, residual renal function, and serum ion examinations, before and after dialysis. Furthermore, levels of sFas and sFasL at baseline had also been measured. We compared demographic data, echocardiographic parameters, liver function, ions, and residual renal function as well as serum sFas and sFasL between the PH and non-PH groups. These parameters were correlated with systolic pulmonary artery pressure (sPAP) using Spearman’s correlation. Moreover, univariate and adjusted logistic regression analyses have also been conducted.

**Results:**

The incidence of PH in the elder dialysis patients was 39.1%. PH populations were demonstrated with significantly higher end-diastolic internal diameters of the left atrium, left ventricle, right ventricle (RV), and pulmonary artery, as well as the left ventricular posterior wall thickness (LVWP; all *p* < 0.05). A higher baseline serum sFas and sFasL levels have also been identified ( *p* < 0.001). They also showed lower fractional shortening and left ventricular ejection fraction (LVEF; *p* < 0.05). Following dialysis, the post-dialysis serum potassium concentration (K^+^) was significantly higher in the PH group ( *p* = 0.013). Furthermore, the adjusted regression identified that ratio of sFas/FasL (OR: 1.587, *p* = 0.004), RV (OR: 1.184, *p* = 0.014), LVPW (OR: 1.517, *p* = 0.007), and post-dialysis K^+^ (OR: 2.717, *p* = 0.040) was the independent risk factors for PH while LVEF (OR: 0.875, *p* = 0.040) protects patients from PH.

**Conclusion:**

The baseline ratio of sFas/sFasL, RV, LVPW, and post-dialysis K^+^ was independent risk factors for PH onset, while LVEF was a protective factor for PH.

## Background

Hemodialysis has been considered as the most used and effective treatments for end-stage renal disease (ESRD) patients (Collins et al., 2001; Zoccali et al., 2004; Li et al., 2014). The regular dialysis has also demonstrated to improve the life time and quality of survival (Himmelfarb and Ikizler, 2010; National Kidney Foundation, 2015; Teitelbaum, 2021). However, long time of dialysis instead of natural renal functions may result in various side effects including the cardiovascular system injury and dysfunction in addition to the side effects of renal failure itself (Collins et al., 2001; O’Lone et al., 2018). The injury on coronary artery and carotid artery have been reported widely (Collins et al., 2001). However, the influence of dialysis on pulmonary circulation (pulmonary hypertension, pulmonary embolism, etc.) has not been widely investigated.

Pulmonary hypertension (PH) has been identified as one of the most common cardiovascular complications associated with various diseases, including cardiovascular diseases and chronic kidney diseases, and particularly in end-stage renal disease (Barbera et al., 2018; Gumus and Saricaoglu, 2020; Walther et al., 2020; Zhang et al., 2020b). PH is characterized with a progressive elevation of pulmonary arterial pressure (PAP; Olschewski et al., 2018). Furthermore, PH is reportedly prevalent in cardiovascular diseases and has also been considered the leading cause of right heart failure, which may be fatal (Li et al., 2014; Reque et al., 2016, 2017).

Moreover, PH has been recognized as a novel threat in treating and preventing ESRD (Etemadi et al., 2012; Shoukat et al., 2014; Zhang et al., 2016). Researchers identified that PH in ERSD is associated with renal failure, which belongs to class V PH (PH with multiple-reason/unclear mechanisms/caused by chronic renal failure with/without dialysis) according to the latest guidelines for the diagnosis and treatment of PH by the European Society of Cardiology (Galie et al., 2016; Barbera et al., 2018). PH incidence was reportedly ranged from 9 to 39% among patients with stage 5 chronic kidney disease while it was various from 8.8 to 68.8% in patients with ESRD who undergo hemodialysis (Reque et al., 2017; Barbera et al., 2018; Olschewski et al., 2018; Zhang et al., 2020b). Additionally, a previous study reported an incidence of up to 42% in patients undergoing peritoneal dialysis (Etemadi et al., 2012).

Several studies have reported on the prevalence of PH in patients with ESRD undergoing maintenance hemodialysis and have investigated the risk factors for PH (Bolignano et al., 2018; Franco, 2018; Rosenkranz et al., 2018). The popularly reported risk factors for PH in ESRD patients were volume overload, hemodynamic instability during hemodialysis, severe anemia, and hyperparathyroidism because of renal failure (Shoukat et al., 2014; Zhang et al., 2016; Nithiya et al., 2020; Walther et al., 2020). Furthermore, the left heart structure and function are also involved in PH onset in patients with ESRD (O’Lone et al., 2018). Additionally, the toxicity of metabolic wastes contributes to PH following renal failure. Blood components and electrolytes have also been associated with PH, which may be attributed to their roles in vascular remodeling *via* vascular endothelial cells and vascular smooth muscle cells (Zhang et al., 2016, 2020b). The apoptosis-related markers Fas and Fas ligand (FasL) have also been reported to be a higher level in the ESRD patients compared with others (Perianayagam et al., 2000). However, they have not been identified in the PH among dialysis patients.

Multiple organs and systems become dysfunctional with the increase of age (Sazlina et al., 2020; Nyirenda, 2021). Other studies have reported that elder age as an independent and in-reversible risk factor for cardiovascular diseases. However, the cardiovascular diseases in elder patients with ESRD who receive hemodialysis have not been identified.

In the past years, accelerated programmed cell death or apoptosis has been demonstrated among patients with chronic renal failure (CRF) which may be caused by uremic toxins (Chen et al., 2021). An increased Fas ligand (FasL, a 40-kd type II integral membrane protein) level in ESRD patients has been reported which is a key regulatory apoptotic pathway of the cell death (Perianayagam et al., 2000). Furthermore, the ratio of sFas/sFasL has also been identified as a marker for vascular dysfunction or vasculopathy (Adly et al., 2016). sFas and/or sFasL has been identified the association with tumor, coronary artery disease, rheumatic disease, and other diseases (Sahinarslan et al., 2012; Adly et al., 2016; Chiloff et al., 2020; Vincent et al., 2020). However, the roles of sFas, sFasL and their ratio in PH with ESRD who undergoing hemodialysis (especially in elder patients) have not been identified. Furthermore, there were few prospective cohort studies reporting on factors and roles of sFas and sFasL in elder patients with renal failure with PH who regularly receive hemodialysis. Thus, we conducted this prospective study with a large population with a cohort at our dialysis centers to determine the predictors for PH especially the predictive roles of sFas and sFasL in addition to other risk factors in PH patients undergoing hemodialysis.

## Materials and Methods

### Study Design and Population

We designed a prospective cohort and longitudinal study with a median follow-up of 5.5 years from August 2013 to December 2020. All together 163 elder patients with ESRD who underwent maintenance hemodialysis were included in the study according to the inclusion criteria and exclusion criteria.

The inclusion criteria were as follows: Patients with ESRD who underwent hemodialysis (<1 month from the first dialysis session). The exclusion criteria were as follows: Patients with one of the following diseases including PH, heart failure, chronic obstructive pulmonary disease, pulmonary embolism, collagen vascular disease, and moderate/severe mitral, or aortic valve diseases. Patients with other conditions that were not suitable for the follow-up were also excluded. We finally included 156 subjects in the analysis. Seven patients were lost to follow-up (three with severe hepatic or cardiac dysfunction, two deaths during hospitalization, one with advanced cancer, and one with severe infection).

Our study was in compliance with the tenets of the Declaration of Helsinki with respect to human research and was approved by the ethics committee of the 940^th^ Hospital of Joint Logistics Support of People’s Liberation Army (PLA) and The Second Medical Center & National Clinical Research Center for Geriatric Diseases, Chinese PLA General Hospital. Each patient was completely informed of the purpose and procedure of this study. All patients provided written informed consent.

**Figure 1:**
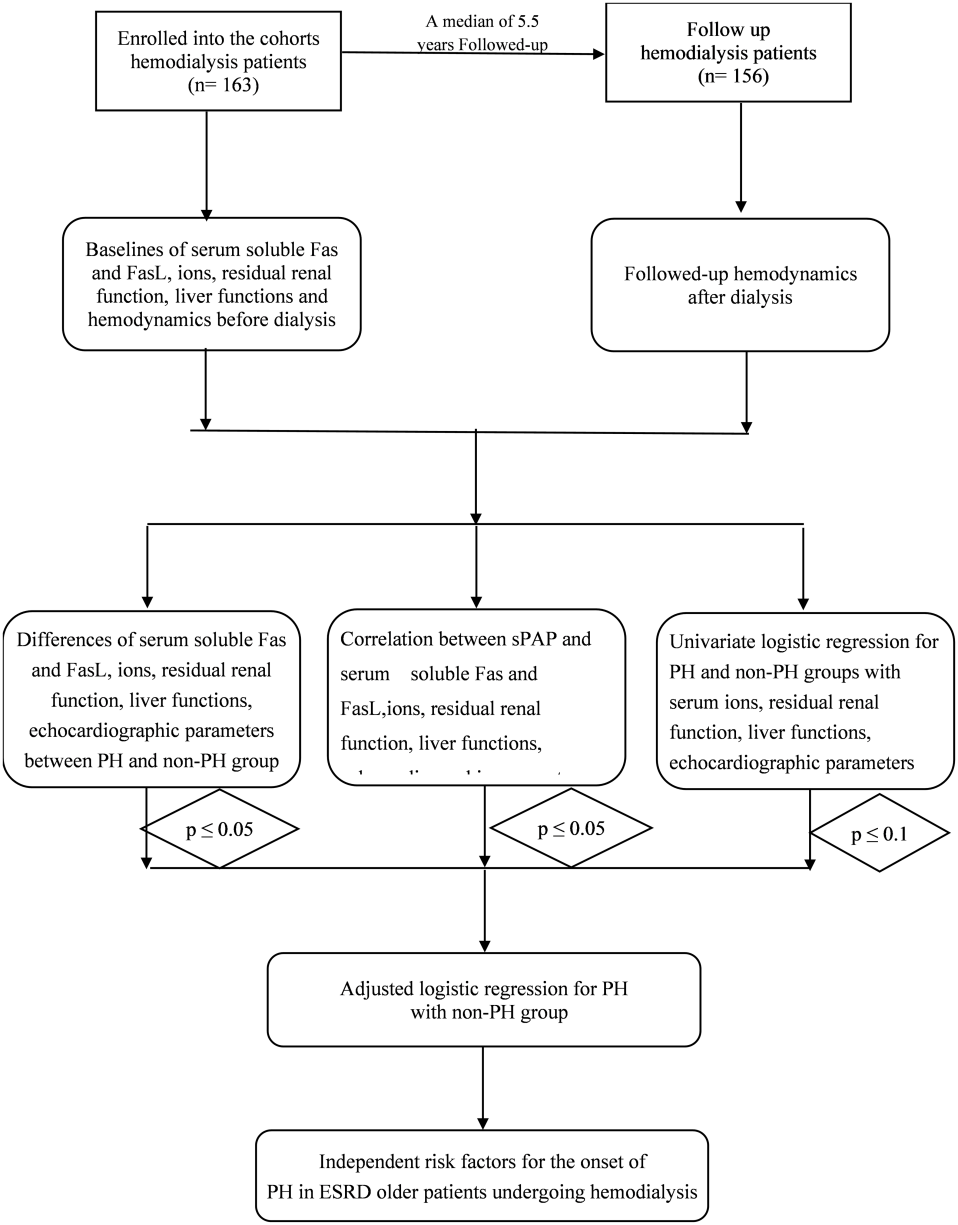
The statistics follow chart of this study.

All patients underwent echocardiography examination following dialysis to avoid the effects of volume overload or fluid overload. Furthermore, they were examined for residual renal function, ion tests before and after dialysis, and liver function examination. Figure 1 depicts the study flow chart.

### Biomarker Variable Determination

We collected venous blood samples from patients following 12-h fasting. The following variables have been examined, respectively: an enzyme assay with Roche Diagnostics GmbH (ARCHITECT i2000SR immunoassay analyzer, Abbott Park, Illinois, United States) was used to test the plasma creatinine (Cr) level, an indirect ion-selective electrode assay (EX-Z, JOKOH, Japan) was used to examine the sodium concentration (Na^+^), tri-azo methods were used to test serum calcium concentration (Ca^2+^), and phosphomolybdate ultraviolet assay with Roche Diagnostics GmbH was used to detected the phosphate concentration (P). Furthermore, we also examined the blood urea nitrogen concentration (BUN) and serum potassium concentration (K^+^; FERENE methods, Beckman AU5821).

We measured the alanine aminotransferase (ALT) and aspartate transaminase (AST) levels using continuous monitoring assays with Reebio kits (Reebio, Beijing, China). Liver function was assessed using the total protein (TP) test by Biuret Method (7600 Series, HITACHI, Tokyo, Japan) and the total albumin (Alb) test by Bromocresol Green method. Chemical oxidation was used to identify the levels of total bilirubin (TBil) and direct bilirubin (BilD). sFasL (Thermo Scientific, China) was measured in serum samples by ELISA according to the manufacturer’s instructions.

All biochemical variables were measured from the blood specimens at the Clinical Laboratory Department of the 940^th^ Hospital of Joint Logistics Support of PLA and the PLA General Hospital.

### Echocardiography Examinations

Trained technicians performed the echocardiographic examinations at both cross-sections by using an ultrasonography workstation (CX50, Philips, United States; probe: S5), based on the American Society of Echocardiography recommendations. The subjects were positioned at a supine position after resting for 10 min. Two-dimensional echocardiography-guided M-mode images were recorded from the standardized views.

The structure and function of the left and right heart were measured and recorded, including the end-diastolic internal diameters of the left and right atria (LA and RA), left and right ventricles (LVDD and RV), and the pulmonary artery (PA).

Moreover, we measured the thickness of the interventricular septum (IVS) and left ventricular posterior wall (LVPW). We also measured the stroke volume (SV), fractional shortening (FS), and ejection fraction (LVEF). We eventually examined tricuspid regurgitation (TR)-related parameters, including the TR area (TRA), TR velocity (the TRV was used to calculate the PAP), and TR pressure.

### Variable Definitions

According to the American Society of Echocardiography, the modified Bernoulli equation using the tricuspid systolic jet was used to calculate the systolic PAP (sPAP):


$$\mathrm{sPAP}=4\times {\left(TRv\right)}^{2}+\mathrm{estimated right atrial pressure}$$

The estimated right atrial pressure was recorded as 5, 10, and 15 mmHg to the right atrium size (normal, mildly enlarged, and significantly enlarged, respectively).

According to the American Society of Echocardiography and other studies, an sPAP >35 mmHg was defined as PH. In contrast, the non-PH group included patients with an sPAP <35 mmHg (Mansour et al., 2010; Rudski et al., 2010).

### Statistical Analyses

The normally distributed continuous variables were expressed as the mean ± standard deviation (SD). We conducted the independent Student’s *t*-test to compare the non-PH and PH groups. The non-normally distributed variables were expressed as medians (25–75%) and were compared using nonparametric tests between the groups.

We conducted univariate logistic regression analyses to screen the independent risk factors for PH. If a variable had a *p* < 0.1, analyzed by univariate logistic regression, it was included in the adjusted logistic regression analyses to identify independent risk factors for PH. All statistical analyses were performed using IBM SPSS Statistics for Windows, version 26.0 (IBM Corp., Armonk, NY, United States).

## Results

### Demographic Information and the Prevalence of PH

A total of 163 patients with ESRD undergoing hemodialysis were included in the cohort.

The mean age and BMI were 68.00 ± 10.51 years and 24.24 ± 3.26 kg/m^2^, respectively. The incidence of PH was 39.1% (61/156). We observed no differences in the demographic data (the age and BMI, *p* > 0.05) between the groups.

The changes of the hemodynamics, remain renal functions, liver functions, and other parameters were reported in Supplementary Table S1. We have found significant changes in the hemodynamics parameters, as well as the sFas and sFasL. The sPAP increased significantly from a median of 28–39 mmHg.

### Differences in the Baseline sFas and sFasL, Echocardiographic, Residual Renal Functions, Liver Function, and Ions Parameters

Among the echocardiographic parameters, the LA and LVDD were significantly higher in the PH group than in the non-PH group (*p* < 0.001).

The LVEF was significantly lower in the PH group (*p* = 0.006). However, the SV did not differ between the groups (*p* > 0.05). The IVS and LVPW thickness also demonstrated a significantly higher level in the PH group, compared to the non-PH group (all *p* < 0.05). Moreover, the baseline RA and RV were higher in the PH group (*p* < 0.001). However, the baseline TR-related parameters were similar between the groups (*p* > 0.05). The PH group demonstrated higher baseline PA and significantly lower FS (Table 1).

**Table 1:** Differences of the baseline hemodynamics, demographic data, renal function, ions, and liver function between PH and non-PH groups.

Baseline Parameters	Overall (*n* = 156)	Non-PH patients (*n* = 95)	PH patients (*n* = 61)	*p*
*Demographic data*				
Age (years)	68.00 ± 10.51	66.20 ± 9.52	69.13 ± 11.55	0.419
BMI (kg/m^2^)	22.24 ± 3.26	21.92 ± 3.37	22.75 ± 3.03	0.117
Female (*n*, %)	86 (55.1%)	47 (49.5%)	39 (63.9%)	0.052
Hypertension (*n*, %)	17 (10.9%)	9 (9.5%)	8 (13.11%)	0.671
Diabetes mellitus (*n*, %)	13 (8.3%)	5 (3.2%)	8 (13.11%)	0.047
*Echocardiographic parameters*				
LA (mm)	36.35 ± 5.11	35.21 ± 4.67	38.10 ± 5.29	<0.001
LVDD (mm)	48.27 ± 6.34	46.80 ± 5.19	50.57 ± 7.29	<0.001
RA (mm)	35.38 ± 4.32	34.21 ± 2.96	37.19 ± 5.37	<0.001
RV (mm)	34.10 ± 3.83	33.12 ± 2.76	35.62 ± 4.70	<0.001
PA (mm)	23.22 ± 2.64	22.75 ± 2.85	23.96 ± 2.11	0.005
IVS (mm)	12.09 ± 2.93	12.00 ± 3.52	12.23 ± 1.66	0.632
LVPW (mm)	11.05 ± 1.49	10.77 ± 1.36	11.49 ± 1.59	0.003
FS (%)	33.59 ± 6.74	34.52 ± 6.63	32.16 ± 6.71	0.033
LVEF (%)	61.92 ± 8.65	63.45 ± 7.39	59.58 ± 9.938	0.006
SV (ml)	72.03 ± 22.25	70.45 ± 22.68	74.94 ± 21.41	0.290
TRA (cm^2^)	1.00 (1.00–2.00)	1.00 (0.88–1.92)	1.50 (1.00–2.25)	0.137
TRV (cm/s)	238.00 (220.00–249.00)	236.50 (220.50–247.00)	240.00 (218.50–253.50)	0.661
∆P (mmHg)	23.00 (20.00–24.50)	22.85 (20.25–24.00)	23.00 (19.00–26.00)	0.888
sPAP (mmHg)	28.00 (25.00–30.00)	27.85 (25.25–29.00)	28.00 (24.00–31.00)	0.876
*Liver functions*				
ALT (U/L)	12.90 (9.02–19.52)	12.20 (8.50–17.8)	13.60 (9.55–21.45)	0.129
AST (U/L)	16.40 (11.02–21.77)	16.60 (11.10–21.80)	15.60 (10.65–21.75)	0.755
TP (g/L)	66.95 (59.55–72.45)	68.10 (59.80–72.60)	65.90 (59.30–71.75)	0.390
Alb (g/L)	39.05 (33.25–43.20)	39.10 (32.90–43.60)	39.00 (34.50–42.95)	0.900
A/G	1.48 (1.27–1.80)	1.45 (1.18–1.75)	1.48 (1.36–1.82)	0.177
TBil (μmoI/L)	10.60 (8.20–28.38)	10.40 (8.00–35.50)	10.80 (8.30–19.05)	0.996
BilD (μmoI/L)	1.70 (1.20–4.65)	1.70 (1.20–6.80)	1.80 (1.20–3.90)	0.826
*Residual renal function*				
Pre-dialysis				
UA (μmoI/L)	464.00 (397.35–529.62)	464.30 (392.60–535.10)	459.40 (407.00–512.30)	0.999
Cr (μmoI/L)	759.61 (531.77–940.08)	759.61 (561.70–937.30)	759.61 (507.20–968.10)	0.695
BUN (μmoI/L)	20.79 (16.35–24.40)	20.74 (16.50–25.31)	20.87 (15.96–23.99)	0.692
Post-dialysis				
UA (μmoI/L)	131.10 (103.50–183.50)	125.40 (102.20–169.0)	150.30 (122.90–196.00)	0.020
Cr (μmoI/L)	318.90 (221.22–423.72)	316.10 (235.40–423.20)	328.00 (192.45–424.50)	0.791
BUN (μmoI/L)	7.42 (5.02–9.76)	7.14 (4.67–9.26)	8.06 (5.24–10.02)	0.104
*Electrolyte parameters*				
Pre-dialysis				
Mg^2+^ (mmol/L)	0.98 ± 0.17	0.99 ± 0.17	0.98 ± 0.16	0.816
K^+^ (mmol/L)	4.75 ± 0.82	4.69 ± 0.80	4.83 ± 0.85	0.318
Ca^2+^ (mmol/L)	2.17 ± 0.21	2.18 ± 0.21	2.15 ± 0.20	0.424
Na^+^ (mmol/L)	138.09 ± 2.73	137.84 ± 2.85	138.47 ± 2.50	0.160
Cl^−^ (mmol/L)	102.90 ± 4.81	102.42 ± 4.70	103.64 ± 4.93	0.125
TCO_2_ (mmol/L)	21.19 ± 3.63	21.35 ± 3.42	20.94 ± 3.94	0.501
P (mmol/L)	1.78 ± 0.56	1.77 ± 0.58	1.79 ± 0.54	0.807
Post-dialysis				
Mg^2+^ (mmol/L)	0.82 ± 0.14	0.82 ± 0.12	0.83 ± 0.15	0.605
K^+^ (mmol/L)	3.46 ± 0.50	3.38 ± 0.43	3.58 ± 0.56	0.013
Ca^2+^ (mmol/L)	2.28 ± 0.14	2.29 ± 0.14	2.28 ± 0.14	0.557
Na^+^(mmol/L)	138.72 ± 3.85	138.79 ± 4.57	138.6 1 ± 2.33	0.770
Cl^−^ (mmol/L)	99.31 ± 3.62	98.98 ± 3.62	99.82 ± 3.59	0.157
TCO_2_ (mmol/L)	25.83 ± 3.81	26.27 ± 3.81	25.14 ± 3.75	0.070
P (mmol/L)	0.83 ± 0.24	0.82 ± 0.26	0.85 ± 0.23	0.581
*Baseline of sFas related variables*				
sFas (pg/ml)	2,117.89 (1,868.38–2,355.50)	1,933.54 (1,676.01–2,197.31)	2,355.49 (2,041.02–3,371.77)	0.014
sFasL (pg/ml)	345.00 (260.25–422.22)	315.99 (260.25–380.01)	398.76 (263.505–461.12)	<0.001
Ratio of sFas/sFasL	6.25 (5.06–8.05)	6.06 (4.96–7.80)	7.36 (5.46–8.53)	0.018

The baseline sFas level has been indicated to be significantly higher [2,355.49 (2,041.02–3,371.77) vs. 1933.54 (1,676.01–2,197.31) pg/ml, *p* = 0.014] in the PH patients than that in the non-PH patients. Similarly, baseline of sFasL level was also higher in the PH patients [398.76 (263.505–461.12) vs. 315.99 (260.25–380.01), *p* < 0.001]. Finally, their ratio sFas/sFasL showed a similar trend in the two groups of populations, PH patients were characterized by higher ratio of sFas/sFasL [7.36 (5.46–8.53) vs. 6.06 (4.96–7.80), *p* = 0.018; Table 1].

We further investigated differences in the baseline residual renal function at both pre- and post-dialysis cross-sections. We observed a significant difference only for uric acid (UA, *p* = 0.020) between the groups. There were no differences in the Cr or BUN between the groups, before or after dialysis (all *p* > 0.05, Table 1).

Pre- and post-dialysis ion parameters were also examined and analyzed. However, K^+^ following dialysis was significantly higher in the PH group (*p* = 0.013). Before dialysis, there were no significant differences in terms of Mg^2+^, K^+^, Ca^2+^, Na^+^, Cl^−^, P, and TCO_2_ between the groups (*p* > 0.05, Table 1).

Liver function tests, including ALT, AST, TP, Alb, A/G, TBil, and DBil, were performed. However, we did not find any differences in terms of liver function between the PH and non-PH groups (all *p* > 0.05, Table 1).

Additionally, the differences of the followed-up data were also shown in Supplementary Table S2.

### Associations Between the sPAP and sFas, sFasL, Demographic, Echocardiographic, Liver Function, Ions, and Residual Renal Function Parameters

We further analyzed the associations between the follow-up sPAP and other baseline parameters. The structures and functions of the heart were significantly correlated with the sPAP, including the LA, LVDD, RA, RV, PA, SV, and LVPW (*p* < 0.05). The LVEF (*p* = 0.041) was negatively associated with the sPAP.

We have found that sPAP was significantly correlated with sFas (*r* = 0.518, *p* < 0.001) while it negatively correlated with sFasL (*r* = −0.709, *p* < 0.001). Regarding the ratio of sFas/sFasL, it was positively correlated with sPAP (*r* = 0.260, *p* = 0.007; Table 2).

**Table 2:** Relationship between sPAP and baseline parameters.

Parameters	Relationship with sPAP	
	*R*	*p* value
*Demographic data*		
Age (years)	0.007	0.946
BMI (kg/m^2^)	0.005	0.960
*Echocardiographic parameters*		
LA (mm)	0.410	<0.001
LVDD (mm)	0.377	<0.001
RA (mm)	0.398	<0.001
RV (mm)	0.396	<0.001
PA (mm)	0.334	<0.001
IVS (mm)	0.141	0.149
LVPW (mm)	0.283	0.003
FS (%)	−0.198	0.041
LVEF (%)	−0.201	0.039
SV (ml)	0.265	0.017
*Residual renal function*		
Pre-dialysis		
UA (μmoI/L)	−0.006	0.955
Cr (μmoI/L)	0.050	0.611
BUN (μmoI/L)	−0.008	0.931
Post-dialysis		
UA (μmoI/L)	0.077	0.432
Cr (μmoI/L)	0.136	0.163
BUN (μmoI/L)	0.184	0.057
*Ions*		
Pre-dialysis		
Mg^2+^ (mmol/L)	0.004	0.971
K^+^ (mmol/L)	0.047	0.629
Ca^2+^ (mmol/L)	−0.081	0.409
Na^+^ (mmol/L)	0.038	0.699
Cl^−^ (mmol/L)	0.004	0.968
TCO_2_ (mmol/L)	−0.097	0.320
P (mmol/L)	0.144	0.140
Post-dialysis		
Mg^2+^ (mmol/L)	0.112	0.248
K^+^ (mmol/L)	0.271	0.005
Ca^2+^ (mmol/L)	−0.008	0.935
Na^+^ (mmol/L)	−0.073	0.452
Cl^−^ (mmol/L)	0.040	0.679
TCO_2_ (mmol/L)	−0.047	0.630
P (mmol/L)	0.107	0.274
*Liver function*		
ALT (U/L)	0.131	0.179
AST (U/L)	0.001	0.990
TP (g/L)	−0.045	0.647
Alb (g/L)	−0.087	0.375
A/G	0.052	0.596
TBil (μmoI/L)	−0.146	0.132
BilD (μmoI/L)	−0.096	0.326
*Baseline of sFas related variables*		
sFas (pg/ml)	0.518	<0.001
sFasL (pg/ml)	−0.709	<0.001
Ratio of sFas/sFasL	0.260	0.007

No significant associations were identified between the sPAP and baseline demographic data, residual renal function (pre- and post-dialysis), and liver function (all *p* > 0.05, Table 2). Similarly, following dialysis only K^+^ levels were significantly associated with the sPAP (*p* = 0.005). Other ions, such as Mg^2+^, Ca^2+^, Na^+^, Cl^−^, P, and TCO_2_, were not correlated with the sPAP, before and after dialysis (all *p* values >0.05, Table 2).

Additionally, Supplementary Tables S3, S4 indicated the associations and the univariate regression results of the followed-up data with PH.

### Logistic Regression for PH Using Baseline sFas, sFasL, Demographic Data, Echocardiographic Parameters, Liver Function Variables, Ions, and Residual Renal Functions

We performed a univariate logistic regression analysis for the PH taking each variable into account to identify potentially independent risk factors.

The age and BMI were not risk factors for PH (*p* > 0.05). We observed a significant association between the structure and function of the heart with PH, including the LA, LVDD, RA, RV, PA, LVPW, FS, and LVEF (*p* < 0.05). Moreover, post-dialysis K^+^ was associated with PH (*p* = 0.027). Furthermore, TCO_2_ was also included in the adjusted regression analysis (*p* = 0.074; Table 3).

**Table 3:** Univariate logistic analysis for PH.

Parameters	*β*	*p* value	OR	95CI%	
				Lower borderline	Upper borderline
*Demographic data*					
Age (years)	0.009	0.416	1.009	0.987	1.032
BMI (kg/m^2^)	0.080	0.118	1.083	0.980	1.197
*Echocardiographic parameters*					
LA (mm)	0.120	0.001	1.128	1.049	1.212
LVDD (mm)	0.103	0.001	1.108	1.044	1.177
RA (mm)	0.208	<0.001	1.231	1.106	1.370
RV (mm)	0.217	<0.001	1.243	1.104	1.399
PA (mm)	0.202	0.008	1.224	1.053	1.423
IVS (mm)	0.027	0.635	0.920	1.027	1.146
LVPW (mm)	0.350	0.005	1.419	1.112	1.811
FS (%)	−0.054	0.038	0.947	0.900	0.997
LVEF (%)	−0.053	0.009	0.948	0.910	0.987
SV (ml)	0.009	0.288	1.009	0.992	1.026
*Residual renal function*					
Pre-dialysis					
UA (μmoI/L)	−0.000	0.766	1.000	0.997	1.003
vCr (μmoI/L)	0.000	0.682	1.000	0.999	1.001
BUN (μmoI/L)	−0.001	0.956	0.999	0.957	1.042
Post-dialysis					
UA (μmoI/L)	0.002	0.205	1.002	0.999	1.005
Cr (μmoI/L)	0.000	0.896	1.000	0.998	1.002
BUN (μmoI/L)	0.091	0.101	1.096	0.982	1.222
*Ions*					
Pre-dialysis					
Mg^2+^ (mmol/L)	−0.228	0.815	0.796	0.118	5.367
K^+^ (mmol/L)	0.201	0.316	1.223	0.825	1.812
Ca^2+^ (mmol/L)	−0.641	0.422	0.527	0.110	2.516
Na^+^ (mmol/L)	0.088	0.161	1.092	0.966	1.234
Cl^−^ (mmol/L)	0.053	0.126	1.055	0.985	1.129
TCO_2_ (mmol/L)	−0.031	0.498	0.970	0.887	1.060
P (mmol/L)	0.071	0.806	1.074	0.608	1.896
Post-dialysis					
Mg^2+^ (mmol/L)	0.618	0.604	1.856	0.179	19.224
K^+^ (mmol/L)	0.879	0.027	2.410	1.103	5.265
Ca^2+^ (mmol/L)	−0.685	0.554	0.504	0.052	4.878
Na^+^ (mmol/L)	−0.012	0.769	0.988	0.909	1.073
Cl^−^ (mmol/L)	0.065	0.157	1.068	0.975	1.169
TCO_2_ (mmol/L)	−0.082	0.074	0.921	0.842	1.008
P (mmol/L)	0.373	0.578	1.452	0.390	5.407
*Liver function*					
ALT (U/L)	0.013	0.405	1.013	0.983	1.044
AST (U/L)	0.008	0.604	1.008	0.979	1.038
TP (g/L)	−0.004	0.641	0.996	0.980	1.012
Alb (g/L)	0.001	0.974	1.001	0.953	1.051
A/G	0.021	0.859	1.021	0.811	1.285
TBil (μmoI/L)	−0.008	0.492	0.992	0.968	1.016
BilD (μmoI/L)	−0.025	0.581	0.976	0.894	1.065
*Baseline of sFas related variables*					
sFas (pg/ml)	0.003	<0.001	1.003	1.002	1.004
sFasL (pg/ml)	0.002	0.068	1.002	1.000	1.004
Ratio of sFas/sFasL	0.241	0.002	1.273	1.095	1.480

Primary screen for the independently risk factors for PH.

In the univariate regression, sFas (*p* < 0.001) and the ratio of sFas/sFasL (*p* = 0.002) were potential risk factors for PH in elder patients who undergoing maintenance hemodialysis (Table 3). However, sFasL showed no significantly predictive roles (*p* = 0.068) while it can also be included into the finally adjusted regression.

We subsequently performed the adjusted logistic regression and identified that ratio of sFas/FasL (OR: 1.587, *p* = 0.004), RV (OR: 1.184, *p* = 0.014), LVPW (OR: 1.517, *p* = 0.007), and post-dialysis K^+^ (OR: 2.717, *p* = 0.040) was the independent risk factors for PH while LVEF (OR: 0.875, *p* = 0.040) protects patients from PH (Table 4).

**Table 4:** Adjusted logistic regressions for PH.

Parameters	*β*	*p* value	OR	95%CI	
				Lower borderline	Upper borderline
RV (mm)	0.169	0.014	1.184	1.035	1.355
LVPW (mm)	0.416	0.007	1.517	1.122	2.050
LVEF (%)	−0.134	0.040	0.875	0.770	0.994
K^+^(mmol/L)	0.999	0.040	2.717	1.046	7.054
Baseline ratio of sFas/sFasL	0.462	0.004	1.587	1.156	2.180

Baseline ratio of sFas/sFasL, RV, LVPW, and post-dialysis K^+^ has been identified to be independent risk factors for PH while baseline of LVEF was an independent protective factor for PH.

At the followed-up section, we have identified that the followed-up ratio of sFas/sFasL, RV, and LVDD was independently associated with PH (Supplementary Table S5).

## Discussion

The incidence of PH in elder patients with ESRD who underwent maintenance hemodialysis was 39.1% at the 5.5 years’ follow-up. Patients with elder age who undergoing maintenance hemodialysis with PH were characterized by higher baseline levels of LA, LV, RA, RV, PA, and LVPW. Moreover, they demonstrated lower baseline levels of FS and LVEF. Following dialysis, the baselines of the UA and K^+^ were also significantly higher in the followed-up PH group, compared to the non-PH group. The adjusted logistic regressions identified four independent risk factors for followed-up PH in elder patients, including baseline RA, LVPW, post-dialysis K^+^, and TCO_2_.

### PH Onset Among Elder Patients With ESRD Receiving Hemodialysis

Pulmonary hypertension has been widely reported and recognized as one of the most frequent complications of numerous diseases, including left heart failure, systemic lupus erythematosus, and renal dysfunction (Reque et al., 2017; Bolignano et al., 2018; Olschewski et al., 2018). PH in patients with ESRD is classified into category V, primarily indicating PH caused by renal dysfunction or unclear mechanisms (Li et al., 2014; Reque et al., 2017; Barbera et al., 2018). Moreover, it is the leading cause of death because of heart failure-associated complications (Reque et al., 2017; Franco, 2018). Therefore, studies on the prevalence of PH or sPAP among patients undergoing maintenance dialysis may provide valuable clues for the management of cardiovascular complications. In the previous study, the investigators found that the hemodialysis patients suffered from PH were characterized with an older age and lower LVEF, which are partly consistent with our present study (Fadaii et al., 2013).

We excluded patients with ESRD and PH previously to avoid a baseline bias by focusing on PH caused by renal failure with dialysis. After a follow-up of 5.5 years, several patients were diagnosed with PH (39.1%). The onset of PH in this study was predominantly due to renal dysfunction and hemodialysis-induced hemodynamic alterations, which may be distinct from the normal physiological state. However, the parameters of hemodialysis, including dialysis ultrafiltration volume and duration, did not show any differences between the PH and non-PH groups or any associations with PH (the aforementioned data are not listed in the tables). Several patients with ESRD had a higher sPAP of 91 mmHg in the follow-up. Such patients with high levels of sPAP were treated with specific drugs or consented to a cardiologist for stand treatment progress.

### Associations Between PH/sPAP and the Baseline Parameters of Residual Renal Function, Liver Function, sFas, sFasL, and Ions

Imbalances of ions, macromolecules, toxins, and nitrogenous wastes (Cr, BUN, and UA) may play critical roles in the development of PH in patients with ESRD. PH is common in patients with ESRD (Shoukat et al., 2014; Zhang et al., 2020b). However, UA was found significantly higher in the PH group, indicating that UA may be a potential risk factor for PH. Neither BUN nor Cr (both pre-dialysis and post-dialysis) differed significantly between the PH and non-PH groups. Similarly, no close associations were identified with the sPAP.

Furthermore, excessive serum ions revealed adverse effects on PH in patients with ESRD, accompanied by disturbances of ions (hyperkalemia, hypermagnesemia, and other statutes). Previous studies have demonstrated that ions contribute to pulmonary vasculature remodeling *via* their roles in biological functions, such as cell proliferation, migration, and differentiation (vascular endothelial cells and smooth muscle cells; Goncharov et al., 2014). Notably, researchers have investigated and reported the roles of K^+^, Ca^2+^, and Mg^2+^ ions (Zhang et al., 2007; Wang et al., 2015; Lv et al., 2019). We analyzed the ion concentration before and after dialysis to identify their associations with PH. Recent investigations of Mg^2+^ have focused on its effect on cAMP response element-binding proteins in neurons (Wu et al., 2018). However, its role in protein synthesis and cardiovascular disease has not yet been studied. Despite Mg^2+^ being identified a potential risk factor for PH in our previous study (Zhang et al., 2020b), we did not observe associations between Mg^2+^ and PH in this study. This difference might be attributed to other strong risk factors of PH, such as the RA and other hemodynamic parameters (Zhang et al., 2020b). Post-dialysis K^+^ is reportedly associated with PH. The patients with PH were initially characterized by a significantly higher level of post-dialysis at baseline. The Spearman’s correlation analysis also revealed a positive correlation between post-dialysis K^+^ and sPAP. In both univariate and adjusted logistic regressions, K^+^ following dialysis was a potential or independent risk factor for PH, thus necessitating basic mechanistic studies to identify the precise roles and mechanisms of K^+^ in the development of PH. In addition, Na^+^ and Cl^−^ did not show any association with PH or sPAP.

It is well known that the Fas and its ligand FasL are markers of cell apoptosis and their combination may participate in the signal transduction of apoptosis (Adly et al., 2016). Furthermore, the soluble Fas and FasL have also been reported in a higher level in CRF (Perianayagam et al., 2000). In the ESRD/CKD patients, both of sFas and sFasL level were significantly higher than that in the control groups which was partly consistent with our results (Perianayagam et al., 2000). In addition, few studies have shown that the sPAP was closely related with sFas and sFasL, which is partly consistent with the previously study (Adly et al., 2016).

We also studied the liver function at baseline. However, the baseline ALT, AST, TP, Alb, A/G, TBil, and DBil did not reveal associations with PH or sPAP. Hence, they were not considered as potential or independent risk factors for PH in further logistic regression analyses.

### PH Was Closely Associated With the Baseline Hemodynamic Parameters

Effects of the hemodynamic state on the clinical outcomes in elder patients with ESRD undergoing maintenance dialysis should be given sufficient attention (Zingerman et al., 2014; Edriss et al., 2018; Chang et al., 2020). Our previous retrospective studies on the prevalence of PH and TR have reported a close association between hemodynamic parameters, such as LVDD and LVEF, and clinical outcomes in the aforementioned patients (Zhang et al., 2016, 2020a,b). In this cohort study, patients with PH had higher levels of LA, LVDD, RA, RV, PA, and LVPW. The aforementioned parameters were also positively correlated with the sPAP. LVEF and FS were significantly lower in the PH group, thus indicating the baseline hemodynamic characteristics may contribute to the onset of PH. Lower baseline left heart functions may indicate the chances of developing PH, necessitating further analysis. To the best of our knowledge, no studies have so far tested the above hypothesis. Moreover, no associated basic research has demonstrated the precise mechanisms behind this phenomenon. The hemodynamics of ESRD elder patients are different from that in the younger patients, which may be caused by the stiffness of artery, the cardiovascular disease such as coronary heart disease, the hypertension, and others. Those diseases have been demonstrated to be product various effects on hemodynamics. Furthermore, the drugs against diseases listed above have also effects on hemodynamics which should also been paid more attentions in the future studies. Despite our efforts to eliminate bias, echocardiogram examinations are easily influenced by fluid overload (volume load; Rudski et al., 2010). Thus, we performed the examinations after dialysis to eliminate bias and to identify the association between hemodynamics and PH. In the further logistic regression analysis, the baseline LA, LVDD, RA, RV, PA, and LVPW were recognized as potential risk factors for PH. In contrast, FS and LVEF were identified as potential protective factors for PH. Following adjustment, only RA and LVPW in hemodynamics were determined as independent risk factors for PH.

### Independent Baseline Risk Factors for PH After 5.5 Years’ Follow-Up Among Elder Patients

We excluded patients with previous PH, left heart-related PH, pulmonary stenosis, and heart failure. Thus, the follow-up PH was primarily caused by renal dysfunction, dialysis, or hemodynamics. First, the baseline heart structure and function demonstrated significant associations with PH or sPAP. However, following adjustment, only RA and LVPW were recognized as independent risk factors for PH. The roles of heart structures in elder hemodialysis patients may be partly attributed from the enlarged heart of elder patients accompanied with cardiovascular diseases (Genctoy et al., 2015; Chang et al., 2020). We determined post-dialysis K^+^ as an independent risk factor for PH. The fluctuation of K^+^ might have affected the vascular endothelial cells and vascular smooth muscle cells in the pulmonary artery, resulted in the remodeling of pulmonary arteries, further causing PH (Yuan et al., 1998; Olschewski et al., 2018). Moreover, K^+^ may play various biological roles in cardiac muscle cells, leading to alterations in the structure of the heart, such as the enlargement of right atria and the thickening of LVPW. This in turn may trigger the progress of secondary PH to heart failure or heart diseases (Smith and Crampin, 2004; Oka et al., 2010). Thus, it can be concluded that the renal function and heart failure might induce PH. In patients undergoing maintenance hemodialysis, dialysis over a prolonged period may cause relative imbalance or unstable state of ions, compared to healthy populations, contributing to the development of PH. Thus, hemodynamic stabilization with or without drugs may be a novel strategy to prevent PH in those undergoing maintenance hemodialysis (Olschewski et al., 2018; Cong et al., 2020).

Furthermore, we found that a lower LVEF at baseline was an independent risk factor for follow-up PH. This result is consistent with previously study that PH dialysis patients were characterized with a lower EF (Fadaii et al., 2013).

It is well known that the Fas/FasL system is recognized as a major pathway for the induction of apoptosis in cells which has been identified in proliferative vasculopathy and endothelial-cell apoptosis. Furthermore, the associations between soluble forms of Fas and FasL in systemic lupus erythematosus, autoimmune disease, and tumor have been widely reported in the past years (Perianayagam et al., 2000; Sahinarslan et al., 2012; Iso et al., 2017; Chiloff et al., 2020).

In consistent with others’ studies on the sickle cell disease patients with asymptomatic for PH (Adly et al., 2016), our mainly findings indicated that the baseline of sFas/sFasL ratio is an independent risk factors for old hemodialysis patients. It has also been demonstrated that sFas/sFasL ratio may be considered as a marker for vascular dysfunction (Adly et al., 2016). Furthermore, sFas was elevated in CRF and ESRD patients (without dialysis) compared to controls (Perianayagam et al., 2000). The sFas level has been founded to be correlated positively with serum creatinine (Perianayagam et al., 2000; Reque et al., 2017). It has also been reported a higher among dialysis patients with cardiovascular disease (Adly et al., 2016). However, the levels and predictive roles of the sFas and sFasL in PH patients who are undergoing hemodialysis especially in the elder patients have not been identified. Therefore, we performed this prospective cohort study to determined levels of sFas and sFasL in PH among elder patients who are undergoing hemodialysis. We found an increased sFas/sFasL ratio among patients with PH complication in elder dialysis patients. After adjusted by other variables, the ratio of sFas/sFasL was still an independent risk factor for the development of PH in hemodialysis patients. Thus, we considered that the ratio of proapoptotic markers sFas and sFasL level may be a biochemical surrogate of PH in hemodialysis patients especially in the elder subpopulations.

### Limitations

Our study may have several limitations that should be addressed in future research. First, our sample size was relatively small. Future studies should consider larger sample size textro address this limitation. Second, despite including some of the frequently used parameters, we should consider other variables. Third, we conducted the baseline examinations at the beginning of the study, only at one or two cross-sections. Dynamic examinations of these parameters may be more precise and valuable to identify the risk factors of PH. The state of nutrition, chronic inflammation, and history are also associated with the onset of PH, which must be investigated in future studies.

## Conclusion

In conclusion, the incidence PH is relatively high in elder patients undergoing maintenance hemodialysis for ESRD. The baselines ratio of sFas/sFasL, RV, LVPW, and K^+^ was independent risk factors for PH onset. In contrast, LVEF was a protective factor for PH. Further basic mechanistic studies are needed to confirm our findings.

## Data Availability Statement

The original contributions presented in the study are included in the article/Supplementary Material, further inquiries can be directed to the corresponding authors.

## Ethics Statement

The studies involving human participants were reviewed and approved by ethics committee of the 940^th^ Hospital of Joint Logistics Support of People’s Liberation Army (PLA) and The Second Medical Center & National Clinical Research Center for Geriatric Diseases, Chinese PLA General Hospital. The patients/participants provided their written informed consent to participate in this study.

## Author Contributions

S-ZB and PY participated in the design of the study. X-HD and S-ZB also drafted the manuscript and performed the statistical analysis. PY reviewed and revised this manuscript critically for important intellectual content. HC performed the echocardiography examination. HC and WZ performed the analysis of sPAP-related measurements. X-HD and XC carried out the collection of demographic data and routine blood examination data. The other laboratory measurements were performed by WZ and JZ. The dialysis-related data were obtained by X-HD, JZ, WZ, and HC. PY and S-ZB contributed equally in his study. All authors contributed to the article and approved the submitted version.

## Funding

This study is supported by the National Natural Science Foundation of China (grants 81901916 and 81801382) and Clinical Program from Xinqiao Hospital, Army Medical University (2016YLC16) and 940^th^ Hospital (2021yxky030).

## Conflict of Interest

The authors declare that the research was conducted in the absence of any commercial or financial relationships that could be construed as a potential conflict of interest.

## Publisher’s Note

All claims expressed in this article are solely those of the authors and do not necessarily represent those of their affiliated organizations, or those of the publisher, the editors and the reviewers. Any product that may be evaluated in this article, or claim that may be made by its manufacturer, is not guaranteed or endorsed by the publisher.

## Abbreviations

Alb: Albumin
A/G: Albumin globulin ratio
ALT: Alanine aminotransferase
AST: Aspartate transaminase
BilD: Bilirubin direct
BUN: Blood urea nitrogen concentration
Ca^2+^: Serum calcium concentration
Cr: Plasma creatinine
ESRD: End-stage renal disease
FS: Fractional shortening
IVS: Interventricular septum
K^+^: Serum potassium concentration
LA: Left atrium end-diastolic internal diameter
LVDD: Left ventricle end-diastolic internal diameter
LVEF: Ejection fraction of left ventricle
LVPW: Left ventricular posterior wall
LYM: Lymphocyte ratio
Na^+^: Sodium concentration
P: Phosphate concentration
PA: Pulmonary artery end-diastolic internal diameter
PAP: Pulmonary arterial pressure
PH: Pulmonary hypertension
RA: Right atrium end-diastolic internal diameter
RV: Right ventricle end-diastolic internal diameter
SV: Stroke volume
sFas: Soluble Fas
sFasL: Soluble FasL
sPAP: Pulmonary artery systolic pressure
TBil: Total bilirubin
TP: Total protein
TR: Tricuspid regurgitation
TRA: TR area
TRV: TR velocity
