# Supplementary material for: Baseline Ratio of Soluble Fas/FasL Predicts Onset of Pulmonary Hypertension in Elder Patients Undergoing Maintenance Hemodialysis: A Prospective Cohort Study
Source: Front Physiol. 2022 Mar 1;13:847172. doi: 10.3389/fphys.2022.847172 (PMC8921550; doi:10.3389/fphys.2022.847172)
Supplement: Supplementary file 2 [file Data_Sheet_2.docx]

Table 1 Changes of hemodynamics, renal function, ions and liver function from the baseline to the followed-up

| Parameters | Baselines | Followed-up | p |
| --- | --- | --- | --- |
| Echocardiographic parameters |  |  |  |
| LA (mm) | 36.35 ± 5.11 | 38.04±5.61 | <0.001 |
| LVDD (mm) | 48.27 ± 6.34 | 49.69 ±7.24 | <0.001 |
| RA (mm) | 35.38 ± 4.32 | 36.85±4.85 | <0.001 |
| RV (mm) | 34.10 ± 3.83 | 35.05 ±4.18 | <0.001 |
| PA (mm) | 23.22 ± 2.64 | 23.88±2.86 | <0.001 |
| IVS (mm) | 12.09 ± 2.93 | 12.22±1.60 | <0.001 |
| LVPW (mm) | 11.05 ± 1.49 | 11.04 ±1.30 | <0.001 |
| FS (%) | 33.59 ± 6.74 | 32.18±6.46 | <0.001 |
| LVEF (%) | 61.92 ± 8.65 | 59.56 ±10.44 | <0.001 |
| SV (ml) | 72.03 ± 22.25 | 70.98 ±16.92 | 0.010 |
| TRA (cm^2^) | 1.00 (1.00-2.00) | 1.50(1.00 -4.00) | 0.035 |
| TRV (cm/s) | 238.00 (220.00-249.00) | 286.00 (249.00 – 339.00) | <0.001 |
| ∆P(mmHg) | 23.00(20.00 - 24.50) | 32.00 (25.00 - 42.00) | <0.001 |
| sPAP(mmHg) | 28.00(25.00-30.00) | 39.00(30.00-53.00) | <0.001 |
| Liver functions |  |  |  |
| ALT (U/L) | 12.90 (9.02 -19.52) | 20.05 (15.67 - 29.15) | <0.001 |
| AST(U/L) | 16.40 (11.02 - 21.77) | 18.30 (12.70 -24.65) | 0.019 |
| TP (g/L) | 66.95 (59.55 -72.45) | 69.15 (64.92 - 77.67) | 0.007 |
| Alb (g/L) | 39.05 (33.25 - 43.20) | 41.05 (38.52 - 44.15) | <0.001 |
| A/G | 1.48(1.27-1.80) | 1.42 (1.21 -1.72) | 0.149 |
| TBil(μmoI/L) | 10.60(8.20 -28.38) | 10.10 (8.40-13.15) | 0.003 |
| BilD(μmoI/L) | 1.70 (1.20 -4 .65) | 1.50 (1.10-2.30) | 0.003 |
| Residual renal function |  |  |  |
| *Pre-dialysis* |  |  |  |
| UA(μmoI/L) | 464.00 (397.35 - 529.62) | 390.00 (287.00 - 498.00) | <0.001 |
| Cr (μmoI/L) | 759.61 (531.77 - 940.08) | 760.40 (542.75 - 1022.50) | 0.324 |
| BUN (μmoI/L) | 20.79 (16.35 - 24.40) | 21.52 (16.52 - 27.39) | 0.169 |
| *Post-dialysis* |  |  |  |
| UA(μmoI/L) | 131.10 (103.50 - 183.50) | 166.50(108.40-200.00) | <0.001 |
| Cr (μmoI/L) | 318.90 (221.22 - 423.72) | 345.40(250.00-443.28) | <0.001 |
| BUN (μmoI/L) | 7.42 (5.02 - 9.76) | 9.36(7.22-11.59) | <0.001 |
| Electrolyte parameters |  |  |  |
| *Pre-dialysis* |  |  |  |
| Mg^2+^ (mmol/L) | 0.98 ± 0.17 | 0.99 ± 0.14 | 0.714 |
| K^+^ (mmol/L) | 4.75 ± 0.82 | 4.82± 0.90 | 0.511 |
| Ca^2+^ (mmol/L) | 2.17 ± 0.21 | 2.22± 0.22 | 0.045 |
| Na^+^(mmol/L) | 138.09 ± 2.73 | 138.83 ±3.03 | 0.020 |
| Cl^-^ (mmol/L) | 102.90 ± 4.81 | 100.83 ±3.93 | <0.001 |
| TCO_2_ (mmol/L) | 21.19 ± 3.63 | 22.01±3.84 | 0.071 |
| P (mmol/L) | 1.78 ± 0.56 | 1.77 ±0.58 | 0.895 |
| *Post-dialysis* |  |  |  |
| Mg^2+^ (mmol/L) | 0.82 ± 0.14 | 0.90 ±0.16 | <0.001 |
| K^+^ (mmol/L) | 3.46 ± 0.50 | 3.85 ±0.45 | <0.001 |
| Ca^2+^ (mmol/L) | 2.28 ± 0.14 | 2.28 ± 0.22 | 0.752 |
| Na^+^(mmol/L) | 138.72 ± 3.85 | 139.49±3.86 | 0.078 |
| Cl^-^ (mmol/L) | 99.31 ± 3.62 | 101.93±3.63 | <0.001 |
| TCO_2_ (mmol/L) | 25.83 ± 3.81 | 23.77 ±3.46 | <0.001 |
| P (mmol/L) | 0.83 ± 0.24 | 1.02 ±0.35 | <0.001 |
| Baseline of sFas related variables | | | |
| sFas(pg/ml) | 2117.89 (1868.38-2355.50) | 2774.44 (2447.57-3085.70) | <0.001 |
| sFasL(pg/ml) | 345.00(260.25-422.22) | 392.26 (295.905-480.06) | <0.001 |
| Ratio of sFas/sFasL | 6.25 (5.06-8.05) | 7.20 (5.82-9.28) | <0.001 |

Table S2 Differences of the followed-up hemodynamics, renal function, ions and liver function between PH and non-PH groups

|  | Non-PH patients (n = 95) | PH patients (n =61) | p |
| --- | --- | --- | --- |
| Echocardiographic parameters |  |  |  |
| LA (mm) | 35.72±4.59 | 41.65±5.14 | <0.001 |
| LVDD (mm) | 46.54 ±4.45 | 54.4±8.11 | <0.001 |
| RA (mm) | 34.48±3.01 | 40.53±4.89 | <0.001 |
| RV (mm) | 33.22 ±4304 | 37.90 ±4.28 | <0.001 |
| PA (mm) | 23.12 ±2.35 | 25.06±3.20 | <0.001 |
| IVS (mm) | 12.10 ± 2.93 | 12.41±1.44 | 0.239 |
| LVPW (mm) | 10.84±1.29 | 11.35±1.28 | 0.019 |
| FS (%) | 33.77 ±4.87 | 29.69±7.77 | <0.001 |
| LVEF (%) | 62.46 ±7.26 | 54.70±13.13 | <0.001 |
| SV (ml) | 67.51 ±15.61 | 78.50±19.03 | <0.001 |
| TRA (cm^2^) | 1.00 (1.00-1.125) | 3.50 (1.10-5.500) | <0.001 |
| TRV (cm/s) | 245.00 (228.00-259.75) | 328.00 (296.00 -369.00) | <0.001 |
| ∆P(mmHg) | 24.00 (21.00-26.25) | 43.00 (35.00 - 54.50) | <0.001 |
| Liver functions |  |  |  |
| ALT (U/L) | 18.10 (12.30 -21.70) | 30.50 (20.25- 42.50) | <0.001 |
| AST(U/L) | 15.60 (11.30- 20.90) | 24.30 (17.00-30.90) | <0.001 |
| TP (g/L) | 73.10 (66.00 -78.70) | 68.00 61.85- 73.55) | 0.001 |
| Alb (g/L) | 42.00 (40.50- 45.40) | 39.70(35.95 - 42.15) | <0.001 |
| A/G | 1.36(1.21-1.68) | 1.53 (1.22-1.795) | 0.094 |
| TBil(μmoI/L) | 10.20 (8.50 -13.20) | 10.10 (8.25-12.95) | 0.886 |
| BilD(μmoI/L) | 1.50 (1.10 -2.30) | 1.50 (1.10-2.40) | 0.813 |
| Residual renal function |  |  |  |
| *Pre-dialysis* |  |  |  |
| UA(μmoI/L) | 355.00 (262.00 - 428.50) | 406.30 (294.37 - 558.42) | 0.013 |
| Cr (μmoI/L) | 664.00 (484.50 - 846.00) | 817.40 (613.00 - 1136.50) | <0.001 |
| BUN (μmoI/L) | 19.60 (14.50 - 25.40) | 23.47 (17.80 - 28.69) | 0.007 |
| *Post-dialysis* |  |  |  |
| UA(μmoI/L) | 150.80 (108.00 - 196.00) | 183.50 (108.50-210.50) | 0.438 |
| Cr (μmoI/L) | 341.40 (250.00- 434.00) | 353.00 (250.00-443.28) | 0.571 |
| BUN (μmoI/L) | 9.37 (7.15 - 11.53) | 9.00 (7.33-11.93) | 0.173 |
| Electrolyte parameters |  |  |  |
| *Pre-dialysis* |  |  |  |
| Mg^2+^ (mmol/L) | 0.99 ±0.14 | 0.99 ±0.14 | 0.610 |
| K^+^ (mmol/L) | 4.86±0.76 | 4.74±1.08 | 0.605 |
| Ca^2+^ (mmol/L) | 2.17±0.23 | 2.30±0.17 | <0.001 |
| Na^+^(mmol/L) | 139.08±2.44 | 138.46 ±3.75 | 0.208 |
| Cl^-^ (mmol/L) | 101.81 ±3.50 | 99.30 ±4.08 | <0.001 |
| TCO_2_ (mmol/L) | 21.25 ±3.78 | 23.19±3.67 | 0.002 |
| P (mmol/L) | 1.88 ±0.56 | 1.60±0.57 | 0.003 |
| *Post-dialysis* |  |  |  |
| Mg^2+^ (mmol/L) | 0.86±0.16 | 0.97 ±0.16 | <0.001 |
| K^+^ (mmol/L) | 3.72 ±0.45 | 4.05±0.38 | <0.001 |
| Ca^2+^ (mmol/L) | 2.26±0.24 | 2.31 ± 0.17 | 0.131 |
| Na^+^(mmol/L) | 139.45 ±3.26 | 139.55±4.67 | 0.871 |
| Cl^-^ (mmol/L) | 102.63 ±3.52 | 100.84±3.55 | 0.002 |
| TCO_2_ (mmol/L) | 24.06 ±2.76 | 23.31 ±4.33 | <0.001 |
| P (mmol/L) | 0.88 ±0.33 | 1.24 ±0.27 | <0.001 |
| Followed-up of sFas related variables | | | |
| sFas(pg/ml) | 2532.93 (2195.58-2878.47) | 3085.70 (2673.73-4417.01) | 0.018 |
| sFasL(pg/ml) | 359.28 (295.90-432.07) | 453.39 (299.60-524.286) | <0.001 |
| Ratio of sFas/sFasL | 6.98 (5.71-8.99) | 8.47 (6.29-9.83) | 0.014 |

Table S3 Relationship between sPAP and followed-up parameters

|  | Relationship with sPAP | |
| --- | --- | --- |
| Parameters | R | p value |
| Echocardiographic parameters |  |  |
| LA (mm) | 0.572 | <0.001 |
| LVDD (mm) | 0.551 | <0.001 |
| RA (mm) | 0.672 | <0.001 |
| RV (mm) | 0.612 | <0.001 |
| PA (mm) | 0.408 | <0.001 |
| IVS (mm) | 0.065 | 0.504 |
| LVPW (mm) | 0.192 | 0.048 |
| FS (%) | -0.400 | <0.001 |
| LVEF (%) | -0.412 | <0.001 |
| SV (ml) | 0.215 | 0.026 |
| Residual renal function |  |  |
| *Pre-dialysis* |  |  |
| UA(μmoI/L) | -0.100 | 0.306 |
| Cr (μmoI/L) | -0.076 | 0.438 |
| BUN (μmoI/L) | -0.053 | 0.588 |
| *Post-dialysis* |  |  |
| UA(μmoI/L) | 0.308 | 0.001 |
| Cr (μmoI/L) | 0.174 | 0.073 |
| BUN (μmoI/L) | 0.209 | 0.031 |
| Ions |  |  |
| *Pre-dialysis* |  |  |
| Mg^2+^ (mmol/L) | 0.089 | 0.371 |
| K^+^ (mmol/L) | 0.071 | 0.464 |
| Ca^2+^ (mmol/L) | 0.592 | <0.001 |
| Na^+^(mmol/L) | -0.090 | 0.356 |
| Cl^-^ (mmol/L) | -0.201 | 0.038 |
| TCO_2_ (mmol/L) | 0.063 | 0.518 |
| P (mmol/L) | 0.049 | 0.617 |
| *Post-dialysis* |  |  |
| Mg^2+^ (mmol/L) | 0.290 | 0.003 |
| K^+^ (mmol/L) | 0.377 | <0.001 |
| Ca^2+^ (mmol/L) | 0.424 | <0.001 |
| Na^+^(mmol/L) | 0.057 | 0.055 |
| Cl^-^ (mmol/L) | -0.252 | 0.009 |
| TCO_2_ (mmol/L) | -0.047 | 0.630 |
| P (mmol/L) | 0.256 | 0.008 |
| Liver function |  |  |
| ALT (U/L) | 0.543 | <0.001 |
| AST(U/L) | 0.498 | <0.001 |
| TP (g/L) | -0.593 | <0.001 |
| Alb (g/L) | -0.374 | <0.001 |
| A/G | 0.260 | 0.007 |
| TBil(μmoI/L) | -0.031 | 0.754 |
| BilD(μmoI/L) | 0.177 | 0.068 |
| Baseline of sFas related variables | | |
| sFas(pg/ml) | 0.518 | <0.001 |
| sFasL(pg/ml) | 0.755 | <0.001 |
| Ratio of sFas/sFasL | 0.366 | <0.001 |

Table S4 Univariate logistic analysis for PH among followed-up data

|  |  |  |  | 95CI% |  |
| --- | --- | --- | --- | --- | --- |
| Parameters | β | p value | OR | Lower borderline | Upper borderline |
| Echocardiographic parameters |  |  |  |  |  |
| LA (mm) | 0.270 | <0.001 | 1.311 | 1.194 | 1.438 |
| LVDD (mm) | 0.208 | <0.001 | 1.231 | 1.145 | 1.323 |
| RA (mm) | 0.405 | <0.001 | 1.500 | 1.311 | 1.715 |
| RV (mm) | 0.408 | <0.001 | 1.503 | 1.302 | 1.736 |
| PA (mm) | 0.293 | <0.001 | 1.340 | 1.151 | 1.559 |
| IVS (mm) | 0.122 | 0.239 | 1.129 | 0.922 | 1.383 |
| LVPW (mm) | 0.298 | 0.022 | 1.348 | 1.044 | 1.739 |
| FS (%) | -0.105 | <0.001 | 0.900 | 0.850 | 0.953 |
| LVEF (%) | -0.077 | <0.001 | 0.926 | 0.892 | 0.962 |
| SV (ml) | 0.038 | <0.001 | 1.039 | 1.017 | 1.060 |
| Residual renal function |  |  |  |  |  |
| *Pre-dialysis* |  |  |  |  |  |
| UA(μmoI/L) | -0.003 | 0.007 | 0.997 | 0.994 | 0.999 |
| Cr(μmoI/L) | -0.002 | <0.001 | 0.998 | 0.997 | 0.999 |
| BUN (μmoI/L) | -0.051 | 0.024 | 0.950 | 0.908 | 0.993 |
| *Post-dialysis* |  |  |  |  |  |
| UA(μmoI/L) | 0.010 | <0.001 | 1.010 | 1.006 | 1.015 |
| Cr (μmoI/L) | 0.004 | 0.002 | 1.004 | 1.002 | 1.006 |
| BUN (μmoI/L) | 0.138 | <0.001 | 1.148 | 1.066 | 1.235 |
| Ions |  |  |  |  |  |
| *Pre-dialysis* |  |  |  |  |  |
| Mg^2+^ (mmol/L) | -0.610 | 0.608 | 0.543 | 0.053 | 5.578 |
| K^+^ (mmol/L) | -0.152 | 0.417 | 0.859 | 0.595 | 1.240 |
| Ca^2+^ (mmol/L) | 2.766 | 0.001 | 15.900 | 3.152 | 80.218 |
| Na^+^(mmol/L) | -0.069 | 0.208 | 0.933 | 0.838 | 1.039 |
| Cl^-^ (mmol/L) | -0.180 | <0.001 | 0.835 | 0.759 | 0.918 |
| TCO_2_ (mmol/L) | 0.141 | 0.003 | 1.152 | 1.050 | 1.263 |
| P (mmol/L) | -0.915 | 0.004 | 0.400 | 0.216 | 0.740 |
| *Post-dialysis* |  |  |  |  |  |
| Mg^2+^ (mmol/L) | 4.505 | <0.001 | 90.476 | 8.747 | 935.832 |
| K^+^ (mmol/L) | 0.947 | 0.020 | 2.579 | 1.160 | 5.734 |
| Ca^2+^ (mmol/L) | 1.165 | 0.133 | 3.206 | 0.702 | 14.649 |
| Na^+^(mmol/L) | 0.007 | 0.870 | 1.007 | 0.926 | 1.096 |
| Cl^-^ (mmol/L) | -0.143 | 0.003 | 0.867 | 0.788 | 0.954 |
| TCO_2_ (mmol/L) | -0.062 | 0.196 | 0.940 | 0.855 | 1.033 |
| P (mmol/L) | 0.373 | 0.578 | 1.452 | 0.390 | 5.407 |
| Liver function |  |  |  |  |  |
| ALT (U/L) | 0.122 | <0.001 | 1.129 | 1.080 | 1.182 |
| AST(U/L) | 0.086 | <0.001 | 1.089 | 1.046 | 1.135 |
| TP (g/L) | -0.246 | <0.001 | 0.782 | 0.707 | 0.864 |
| Alb (g/L) | -0.075 | 0.001 | 0.927 | 0.888 | 0.968 |
| A/G | 0.875 | 0.062 | 2.398 | 0.958 | 6.006 |
| TBil(μmoI/L) | -0.001 | 0.983 | 0.999 | 0.933 | 1.070 |
| BilD(μmoI/L) | -0.016 | 0.866 | 0.984 | 0.819 | 1.183 |
| Baseline of sFas related variables | | | | | |
| sFas(pg/ml) | 0.002 | 0.068 | 1.002 | 1.000 | 1.004 |
| sFasL(pg/ml) | 0.002 | <0.001 | 1.002 | 1.002 | 1.003 |
| Ratio of sFas/sFasL | 0.209 | 0.002 | 1.233 | 1.082 | 1.405 |

Table S5 Adjusted logistic regressions for PH

| Parameters | β | p value | OR | 95%CI | |
| --- | --- | --- | --- | --- | --- |
|  |  |  |  | Lower borderline | Upper borderline |
| LVDD (mm) | 0.107 | 0.019 | 1.114 | 1.018 | 1.219 |
| RV (%) | 0.250 | 0.001 | 1.283 | 1.101 | 1.496 |
| Followed-up ratio of sFas/sFasL | 0.243 | 0.014 | 1.275 | 1.050 | 1.548 |

In another adjusted regression model, K^+^ (β = 1.727, p = 0.005; OR = 5.624;95%CI1.676- 18.878) was also identified as the associated factors for PH.
