# Supplementary material for: Baseline Ratio of Soluble Fas/FasL Predicts Onset of Pulmonary Hypertension in Elder Patients Undergoing Maintenance Hemodialysis: A Prospective Cohort Study
Source: Front Physiol. 2022 Mar 1;13:847172. doi: 10.3389/fphys.2022.847172 (PMC8921550; doi:10.3389/fphys.2022.847172)
Supplement: Supplementary file 3 [file Presentation_1.PPTX]

## Slide 1
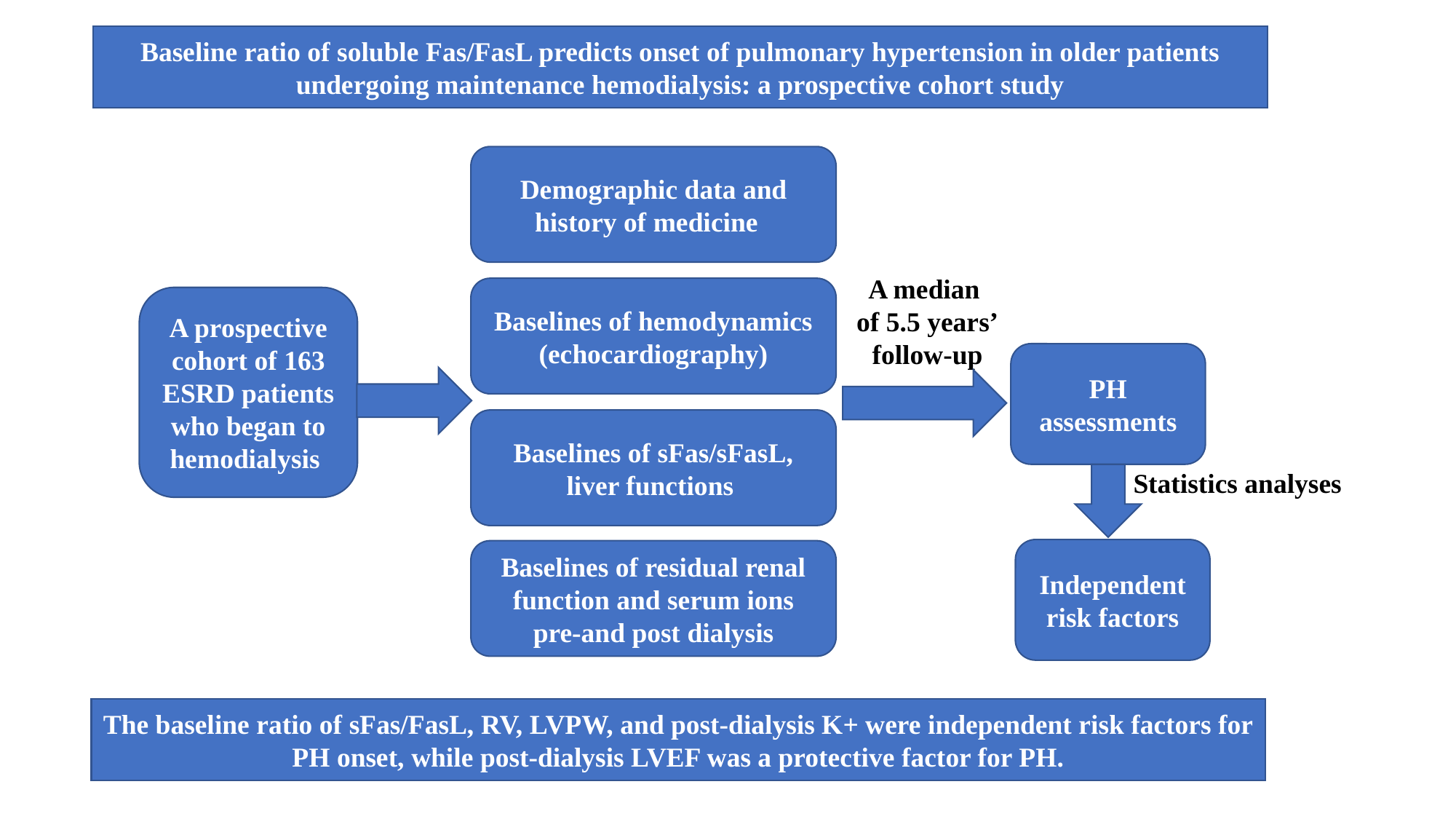

Baseline ratio of soluble Fas/FasL predicts onset of pulmonary hypertension in older patients undergoing maintenance hemodialysis: a prospective cohort study
Demographic data and history of medicine
A median
of 5.5 years’
follow-up
Baselines of hemodynamics
(echocardiography)
A prospective cohort of 163 ESRD patients who began to hemodialysis
PH assessments
Baselines of sFas/sFasL, liver functions
Statistics analyses
Independent risk factors
Baselines of residual renal function and serum ions pre-and post dialysis
The baseline ratio of sFas/FasL, RV, LVPW, and post-dialysis K+ were independent risk factors for PH onset, while post-dialysis LVEF was a protective factor for PH.
